# Plasma miRNA profiles associated with stable warfarin dosage in Chinese patients

**DOI:** 10.7717/peerj.9995

**Published:** 2020-10-13

**Authors:** Li Zhao, Jin Wang, Shaoxin Shi, Yuan Wu, Jumei Liu, Shiwei He, Yue Zou, Huabin Xie, Shengxiang Ge, Huiming Ye

**Affiliations:** 1 School of Medicine, Xiamen University, Xiamen, China; 2 Department of Clinical Laboratory, Women and Children’s Hospital, School of Medicine, Xiamen University, Xiamen, China; 3 National Institute of Diagnostics and Vaccine Development in Infectious Diseases , School of Public Health, Xiamen University, Xiamen, China; 4 Department of Clinical Laboratory, Haicang Hospital, Xiamen, China.; 5 Xiamen Cardiovascular Hospital, School of Medicine, Xiamen University, Xiamen, China

**Keywords:** Warfarin, MicroRNAs, Bioinformatic analysis, Stable dosage

## Abstract

**Background:**

We used bioinformatic analysis and quantitative reverse transcription polymerase chain reaction (RT-qPCR) assays to investigate the association between plasma microRNAs (miRNAs) and stable warfarin dosage in a Chinese Han population.

**Methods:**

Bioinformatics analysis was used to screen out potential warfarin dose-associated miRNAs. Three plasma miRNAs were validated in 99 samples by RT-qPCR. Kruskal–Wallis test and multivariate logistic regression were used to compare differences in plasma miRNAs expression levels between three warfarin dosage groups.

**Results:**

There were significant between-group differences among the three dose groups for hsa-miR-133b expression (*p* = 0.005), but we observed an “n-shaped” dose-dependent curve rather than a linear relationship. Expression levels of hsa-miR-24-3p (*p* = 0.475) and hsa-miR-1276 (*p* = 0.558) were not significantly different in the multivariate logistic regression.

**Conclusion:**

miRNAs have received extensive attention as ideal biomarkers and possible therapeutic targets for various diseases. However, they are not yet widely used in precision medicine. Our results indicate that hsa-miR-133b may be a possible reference factor for the warfarin dosage algorithm. These findings emphasize the importance of a comprehensive evaluation of complex relationships in warfarin dose prediction models and provide new avenues for future pharmacogenomics studies.

## Introduction

Long-term anticoagulation therapy for thromboembolic diseases remains an important clinical issue (Chinese Society of Cardiology, 2013). The oral coumarin anticoagulant warfarin has been used worldwide for anti-thromboembolic diseases such as deep vein thrombosis and atrial fibrillation (Jauch et al., 2013; Ruff et al., 2014). However, warfarin has two main issues that must be addressed: a narrow therapeutic index and a high degree of inter-individual variability in optimal dosing (between 0.6 and 15.5 mg/day), which easily cause blood and thrombus side effects (Kamali & Wynne, 2010; Rieder et al., 2005). Environmental, genetic, and epigenetic factors all influence drug reactivity (Tavares, Marcatto & Santos, 2018). Genome wide association studies have demonstrated significant correlations between warfarin dose and gene polymorphisms (e.g., CYP2C9, VKORC1, and CYP4F2, etc.) (Kaye et al., 2017; Takeuchi et al., 2009). However, the 40–60% accuracy of the current warfarin dosage algorithm is not sufficient for clinical demands (Hu & Yu, 2013; Sagreiya et al., 2010). In the age of precision medicine, predicting the dose of drugs and reducing side effects has become a vital issue needs to be solved urgently.

MicroRNAs (miRNAs) are a class of endogenous non-coding small RNA molecules (Bartel, 2004). They are considered the most abundant type of gene expression regulators and known as the “trimmer of life” (He & Hannon, 2004). Circulating miRNAs can be stable in various biofluids, which led them to be widely studied as ideal biomarkers and possible therapeutic targets in a variety of neurodegenerative diseases (Lukiw, Zhao & Cui, 2008) and tumors (Berindan-Neagoe et al., 2014). In liver samples, hsa-miR-133a was inversely correlated with vitamin K 2,3-epoxide reductase complex subunit 1 (VKORC1) mRNA levels (Pérez-Andreu et al., 2013). That result was consistent with the bioinformatics prediction of two highly conserved binding sites for hsa-miR-133 and hsa-miR-137 on the VKORC1 gene (Shomron, 2010). These studies suggested that miRNAs may affect warfarin treatment by regulating VKORC1, which needs further verification. In recent years, many pieces of researches indicated that miRNAs polymorphisms (Ciccacci et al., 2015; Hosseindokht et al., 2020), and SNPs in miRNAs binding sites (Hosseindokht et al., 2019) could also affect the expression of warfarin target genes and further influence drug response. On the other hand, Rad, Mazaheri & Firozabadi (2018) and Jia et al. (2015) suggested that miRNAs could be also affected by warfarin.

The existing evidence suggests that there are complex interactions between warfarin and miRNAs. However, no study has assessed the profile of miRNAs associated with stable warfarin dosage or specific correlation between them. We examined the differential expression profiles of plasma miRNAs related to warfarin dose, with the goal of identifying new biomarkers for further improving the warfarin dosage algorithm. We screened out several of the most relevant miRNAs by bioinformatics analysis and identified them as biomarkers for warfarin precision medicine in clinical samples. Herein, we present the first description of the association between plasma miRNAs and different stable warfarin dosages in Chinese patients.

## Methods

### Study design

We enrolled 99 patients on different stable doses of warfarin under treatment at the Cardiovascular Hospital (School of Medicine, Xiamen University). A nested case-control study was designed to identify plasma miRNAs as surrogate biomarkers for warfarin precision medicine. Bioinformatics analysis revealed the key genes related to warfarin dose, then a set of warfarin dose-related miRNAs were predicted using the miRNAs-target gene prediction databases. Our predictions were verified using stem-loop quantitative reverse transcription and hydrolysis probe-based RT-qPCR assays in 99 patients. Plasma collection and diagnosis were carried out following standard procedures. Written informed consent was obtained from all patients before study participation. The Ethics Committee of the School of Medicine, Xiamen University, China approved the protocol (IRB approval number: KY2014001), and the study was conducted following the principles of the Declaration of Helsinki.

### Sample collection and patient characteristics

Patients were eligible if they were 18 years of age or older and on long-term warfarin therapy. Other eligibility criteria included: (1) Chinese Han ethnicity, (2) baseline liver and kidney function test data and international normalized ratio (INR) results available, (3) not taking other anticoagulant drugs, (4) not undergoing chemotherapy, (5) no family history of alcoholism, and (6) no history of chronic liver failure or other liver diseases.

According to the consensus of domestic experts (Chinese Society of Cardiology, 2013), the standard targeted INR is 2.0–3.0, but it is 2.5–3.5 for patients with acute valve replacement and two-valve implantation. A stable dose is defined as the mean dose of three consecutive measurements taken at intervals of more than one week to determine the same daily dose to maintain INR within the target range. The study subjects were divided into low-dose (≤2 mg/day), medium-dose (2 to 4 mg/day), and high-dose (≥4 mg/day) groups. Among the 99 patients, 30 (30.3%), 40 (40.4%), 29 (29.3%) were classified as low-, medium-, and high-dose, respectively. patient demographic and clinical characteristics were obtained from the Laboratory Information System or a follow-up telephone call (Table 1).

### Bioinformatics analysis

PharmGKB is a pharmacogenetics and pharmacogenomics database detailing how genetic variation affects drug response. It provides a theoretical basis for research into personalized medicine and pharmacogenomics based on the assumption that differential expression of key pathway genes may impact dose requirements. We screened out key genes that were supported at least two annotations in PharmGKB.

For functional enrichment analysis of predicted genes, GO (Gene Ontology) and KEGG (Kyoto Encyclopedia of Genes and Genomes) pathway enrichment analyses were conducted using the DAVID (Database for Annotation, Visualization, and Integration Discovery, https://david.ncifcrf.gov/summary.jsp). Parameters for the enrichment analysis were as follows: *p* < 0.05, gene count ≥2, top 5.

**Table 1:** Demographic and clinical features of patients with different warfarin dosage.

Variable	Low dose(30)		Medium dose(40)	High dose(29)	*P*-values
Age (years), mean ± SD	63.1 ± 9.382		62.35 ± 9.590	58.72 ± 8.179	0.148
Gender (M%)	14(46.67)		24(60.00)	9(31.03)	0.246
High, mean ± SD	161.787 ± 5.995		163.115 ± 4.933	164.269 ± 5.948	0.184
Weigh, mean ± SD	64.273 ± 6.957		65.190 ± 9.092	67.797 ± 8.517	0.307
Smoking, n (%)	5(16.67)		5(12.50)	2(6.90)	0.323
Drinking, n (%)	4(13.33)		3(7.5)	1(3.45)	0.236
Combination Use Of Amiodarone, n (%)	5(16.67)		3(7.5)	1(3.45)	0.113
Warfarin Stable Dose(mg per day), mean ± SD	1.625 ± 0.278		2.956 ± 0.484	5.086 ± 0.732	0.00^*^
Indications, n (%):					0.080
Atrial fibrillation	13(43.33)		17(42.50)	7(24.14)	
Valve Replacement		8(26.67)		9(22.50)	9(31.03)	
Valvular Heart Disease	7(23.33)		9(22.50)	8(27.59)	
Coronary Heart Disease	2(6.67)		4(10.00)	3(10.34)	
Others	0(0.00)		1(2.50)	2(6.90)	

**Notes.**

* Significant association with *p* < 0.05.

**Table 2:** Prediction databases and websites of miRNAs.

Software	Website
miRanda	http://www.microrna.org
TargetScan	http://www.targetscan.org
DIANA	http://www.microrna.gr
RNA-22GUI	http://cm.jefferson.edu/rna22
PITA	http://genie.weizmann.ac.il/pubs/mir07/mir07_data.html
RNAhybrid	http://bibiserv.cebitec.uni-bielefeld.de/rnahybrid
miRWalk3.0	http://mirwalk.umm.uni-heidelberg.de/
miRDB	http://www.mirdb.org/miRDB/

Five miRNAs prediction sites (miRanda, miRDB, miRWalk, RNA22, TargetScan) were used to identify relevant miRNAs that target warfarin key genes. The final result set was validated in subsequent RT-qPCR assays. The commonly used miRNAs prediction sites were summarized in Table 2.

### miRNAs isolation and quantification

All patients in this study had peripheral blood drawn at study enrollment. Blood samples were collected in commercial vacuum blood collection tubes containing 3.2% sodium citrate and centrifuged at 3500 rpm for 10 min to obtain plasma. After 15-min high-speed centrifugation at 13300 rpm at room temperature to remove cell debris, the supernatant plasma was recovered and stored at −80 °C until analysis.

To assess the expression level of the targets and minimize operational bias, cel-miR-39 was used as an external reference. Before miRNAs extraction, 13.2 pg cel-miR-39 was added to each plasma sample, followed by the same extraction and amplification process as the targets. MiReasy Serum/Plasma kits (cat. #217184; Qiagen, Hilden, Germany) were used to extract miRNAs from 200 µl plasma (including 13.2 pg cel-miR-39) with column extraction, then 25 µl elution buffer was added. Next, we carried out TaqMan probe-based RT-qPCR assays with two commercial kits on the Applied Biosystems 7500 Sequence Detection System (Applied Biosystems, Foster City, CA, USA): TaqMan™ MicroRNA Reverse Transcription Kit based on the stem–loop primer method (cat. #4366597; Applied Biosystems) for the reverse-transcription step and TaqMan universal master MixII, no UNG (cat. #4440040; Applied Biosystems) for qPCR assays. All reactions, including the no-template controls, were run in duplicate. All operations were carried out following standard procedures.

### Statistical analyses

All statistical analyses and plots were conducted using IBM SPSS Statistics 25 (IBM Corp, Armonk, NY, USA). Baseline characteristics were compared by the Kruskal–Wallis test (for continuous variables) or Fisher’s exact test (for categorical variables) to analyze differences among the three groups. The relative expression level of target miRNAs (denoted by ΔΔCt) was corrected by cel-miR-39 (ΔCt _sample_= Ct_target_ −Ct_*cel*−*miR*−39_) and the reference sample (ΔΔCt _sample_ = ΔCt_sample_ − ΔCt_ref._ _sample_) and then used as the data for subsequent statistical analyses. We used Kruskal–Wallis test and multivariate logistic regression to compare differences in plasma miRNAs relative expression level among the three warfarin dosage groups. For all tests, two-tailed *p* < 0.05 was considered statistically significant.

## Results

### Patient characteristics

From April 2018 through November 2018, we enrolled a total of 99 subjects. We recorded the baseline demographics and clinical characteristics of the patients receiving stable warfarin treatment. There were no significant differences in baseline characteristics among the low-, medium- and high-dose groups (Table 1).

### Identification of key genes

Overall, 52 relevant genes were identified in the PharmGKB database, of which 31 genes were supported by at least two annotations (Table 3). Three or more evidences support the CYP2C9, VKORC1, CALU, CYP2C19, CYP1A1, CYP4F2, EPHX1, GGCX, and PROS1 genes. PharmGKB clinical annotations (CAs) provide information about variant-drug pairs based on variant annotations in the database. Scientific curators manually review variant annotations and create genotype-based summaries describing the variants’ phenotypic impacts (Whirl-Carrillo et al., 2012). CAs were divided into four levels according to the strength of the evidence, with CA^1^ meeting the highest criteria. VKORC1, CYP2C9, and CYP4F2 genes were rated as CA^1^, and their effects on warfarin dosage have been widely reported (Tavares, Marcatto & Santos, 2018).

**Table 3:** Genes associated with warfarin and supporting evidences.

Gene symbol		Gene title		Annotation evidence		
ABCB1	ATP binding cassette subfamily B member 1				VA PW	
APOE	apolipoprotein E				CA^3^ VA	
ASPH	aspartate beta-hydroxylase				CA^3^ VA	
CALU	calumenin				CA^2^ VA PW	
CYP1A1	cytochrome P450 family 1 subfamily A member 1				CA^3^ VA PW	
CYP1A2	cytochrome P450 family 1 subfamily A member 2				VA PW	
CYP2C18	cytochrome P450 family 2 subfamily C member 18				VA PW	
CYP2C19	cytochrome P450 family 2 subfamily C member 19				CA^3^ VA PW	
CYP2C9	cytochrome P450 family 2 subfamily C member 9				DLCA^1^VA PW	
CYP3A4	cytochrome P450 family 3 subfamily A member 4				VA PW	
CYP4F2	cytochrome P450 family 4 subfamily F member 2				CA^1^ VA PW	
DDHD1	DDHD domain containing 1				CA^3^ VA	
DNMT3A	DNA methyltransferase 3 alpha				CA^3^ VA	
EPHX1	epoxide hydrolase 1				CA^3^ VA PW	
FPGS	folylpolyglutamate synthase				CA^3^ VA	
GGCX	gamma-glutamyl carboxylase				CA^2^ VA PW	
HNF4A	hepatocyte nuclear factor 4 alpha				CA^3^ VA	
NEDD4			neural precursor cell expressed, developmentally down-regulated 4, E3 ubiquitin-protein ligase				CA^3^ VA	
NQO1	NAD(P)H quinone dehydrogenase 1				CA^3^ VA	
NR1I3	nuclear receptor subfamily 1, the group I, member 3				CA^3^ VA	
POR	cytochrome p450 oxidoreductase				CA^3^ VA	
PROC	protein C, an inactivator of coagulation factors Va and VIIa				DL VA	
PROS1	protein S (alpha)				DL VA PW	
PRSS53	protease, serine 53				CA^3^ VA	
STX1B	syntaxin 1B				CA^3^ VA	
STX4	syntaxin 4				CA^3^ VA	
THBD	thrombomodulin				CA^3^ VA	
UGT1A1	UDP glucuronosyltransferase family 1 member A1				CA^3^ VA	
VKORC1	vitamin K epoxide reductase complex subunit 1				DL CA^1^ VA PW	
F2	coagulation factor II				VA PW	
F7	coagulation factor VII				VA PW	

**Notes.**

DL Drug Label Annotation CA Clinical Annotation CA1 level 1 of Clinical Annotation VA Variant Annotation PW Pathway

**Figure 1:**
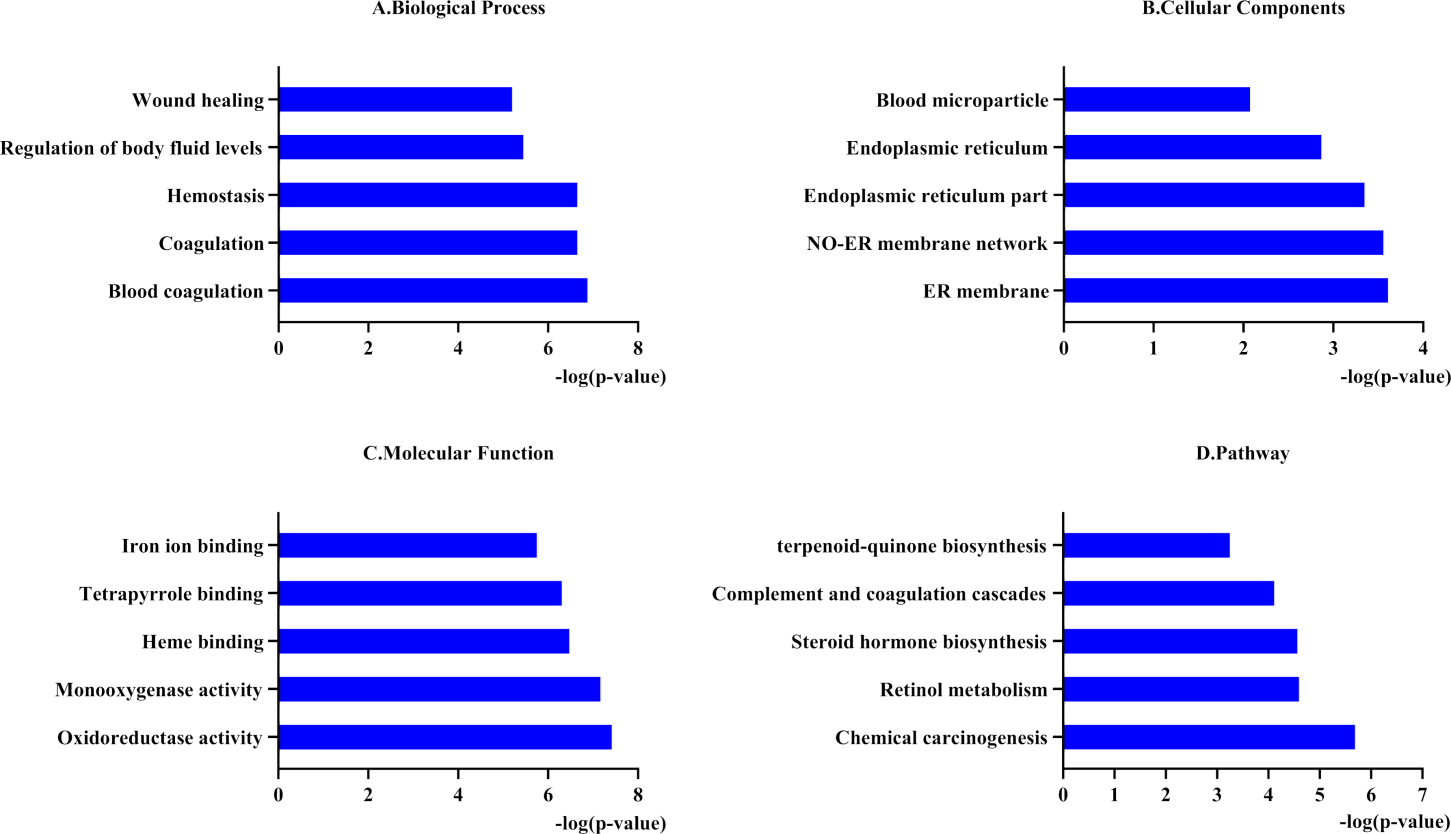
Functional enrichment analysis of 31 predicted genes. (A, B, C) The biological process, cellular components, and molecular function category in GO analysis. Each category is sorted by p-value to display the first five items. (D) KEGG pathway analysis of biological process analysis of 31 predicted genes.

To better understand the functions of the 31 genes predicted by PharmGKB, we performed GO enrichment and KEGG pathway analyses. In the GO enrichment analysis, the predicted genes were significantly enriched in biological processes related to blood coagulation, hemostasis, and body fluid regulation. KEGG pathway analysis revealed that the predicted genes were mainly enriched in chemical carcinogenesis, retinol metabolism, and complement and coagulation cascades (Fig. 1).

According to the PharmGKB “PW” annotation and the results of pathway enrichment analyses, we divided the 31 genes into two groups: pharmacokinetics and pharmacodynamics. We followed conditions to select the key genes of each pathway for subsequent miRNAs prediction. The conditions were as follows: (1) at least two supporting annotations, and (2) top 2 levels for “CA” or the widely reported genes related to warfarin dosage. Finally, we obtained three key genes related to pharmacokinetics: CYP1A1 (Luo et al., 2017), CYP2C9, and CYP2C19 (Zhang et al., 2016), and four key genes in pharmacodynamics: VKORC1, GGCX, CYP4F2, and CALU.

### Prediction of miRNAs targeting key genes

**Figure 2:**
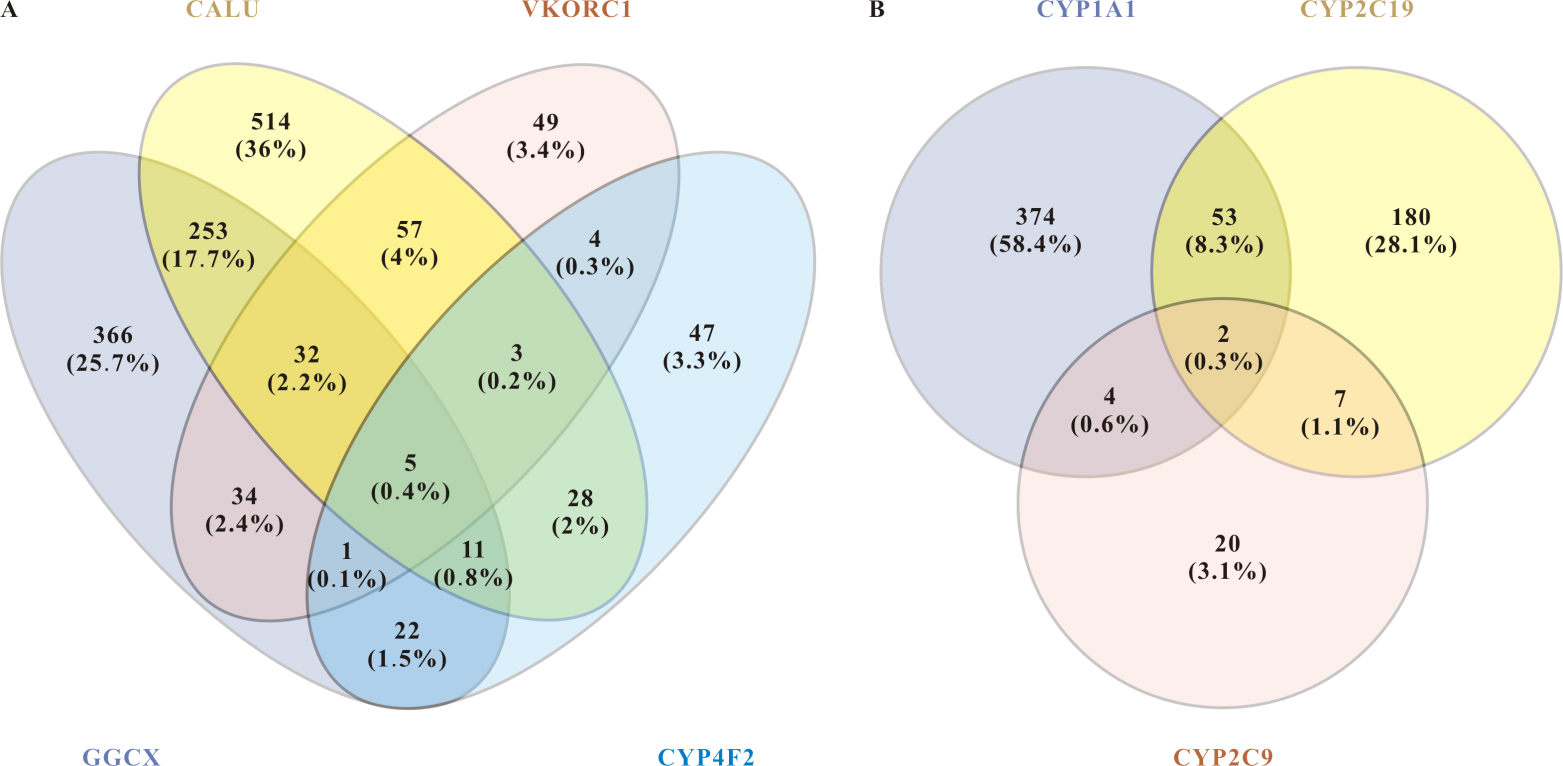
miRNAs related to pharmacodynamics and pharmacokinetics of warfarin. (A) Venn diagram of the related miRNAs to the pharmacodynamics of warfarin. The target mining function of the miRWalk database prediction results show that five miRNAs are significantly associated with these four genes. They are hsa-miR-937-5p, hsa-miR-4700-5p, hsa-miR-1276, hsa-miR-500a-3p, and hsa-miR-940. (B) Venn diagram of the related miRNAs to the pharmacokinetics of warfarin. For warfarin pharmacokinetics, hsa-miR-211-3p and hsa-miR-6515-5p were the most relevant.

Commonly used miRNAs prediction databases include miRanda, miRDB, miRWalk, RNA22, TargetScan. MiRWalk provides more comprehensive predictions because it integrates two predicted databases (TargetScan and miRDB) and one validated database (miRTarBase). We used miRWalk as the primary tool, with the others as supplements. We inputted the 31 differential genes and screened out miRNAs with *p* < 0.05. We followed three principles for further screening: (1) binding with 3′ UTR, CDS or 5′ UTR, the more binding sites, the higher score the miRNA was; (2) consistent output in at least two databases and. (3) miRNAs that can target at least three key genes of warfarin. They were summarized in Table 4. The key genes related to drug metabolism and drug effects obtained from the above results were used for further prediction (Fig. 2).

**Table 4:** The key miRNAs related to 31 genes and their target genes.

NO.	miRNAs	Related genes
1	has-miR-7106-5p	CYP4F11, HNF4A, NQO1, DDHD1, DNMT3A
2	has-miR-6780-5p	THBD, GGCX, CYP4F11, NQO1
3	has-miR-6769a-5p	VDR, STX1B, HNF4A
4	has-miR-6742-3p	STX1B, CYP1A1, DDHD1, VDR
5	has-miR-4728-3p	CALU, DDHD1, NQO1, CYP4F11
6	has-miR-8085	STX1B, GATA4, CALU, DNMT3A
7	has-miR-30b-3p	CYP4F11, NQO1, THBD
8	has-miR-3689b-3p	GGCX, NQO1, THBD
9	has-miR-3689c	GGCX, NQO1, THBD
10	has-miR-4447	GGCX, STX1B, HNF4A
11	has-miR-4717-5p	DDHD1, GATA4, GGCX
12	has-miR-6715b-5p	CYP3A4, STX1B, THBD
13	has-miR-6721-5p	EPHX1, VDR, STX1B
14	has-miR-6870-5p	GGCX, F7, CYP4F11
15	has-miR-6884-5p	NQO1, VDR, HNF4A
16	has-miR-7110-5p	VDR, EPHX1, THBD
17	has-miR-7847-3p	DDHD1, CYP3A4, STX1B
18	has-miR-92a-2-5p	VDR, GGCX, STX1B

About warfarin pharmacodynamics, we obtained five of the most relevant miRNAs: hsa-miR-937-5p, hsa-miR-4700-5p, hsa-miR-1276, hsa-miR-500a-3p, and hsa-miR-940. For warfarin pharmacokinetics, hsa-miR-211-3p and hsa-miR-6515-5p were the most relevant. Warfarin exerts its anticoagulant effect as an inhibitor of VKOR, and it is metabolized in the liver by the enzymes encoded by the cytochrome P450 (CYP) superfamily of genes. The CYP superfamily is responsible for >75% of phase drug metabolism in the liver, so other prescriptions taken by enrolled patients may influence warfarin pharmacokinetics-related miRNAs. Moreover, as a direct target of warfarin, VKORC1 plays a leading role in individualized warfarin dosage. We therefore selected VKORC1, which highly related to warfarin dose, for further screening and verification. We inputted “VKORC1” and screened out miRNAs with *p* < 0.05. miRNAs predicted by at least two databases were considered as VKORC1-related miRNAs. Next, we used miRWalk to reversely predict the targeted genes of these miRNAs, finally screening out 20 miRNAs related to VKORC1 (Table 5). Notably, hsa-miR-1276 was the only one with more than one binding site on VKORC1 mRNA. Synthesizing the above results and information in the literature (e.g., essential functions reported, expression level, etc.), we finally screened out the top three highest score miRNAs: hsa-miR-24-3p(MIMAT0000080), hsa-miR-133b(MIMAT0000770), and hsa-miR-1276(MIMAT0005930).

**Table 5:** The miRNAs related to VKORC1 gene.

miRNAs	*P*-Values	Target genes
hsa-miR-644	0.0196	VKORC1
hsa-miR-612	0.0196	VKORC1, DNMT3A, NR1I3, DDHD1, FPGS
hsa-miR-330-3p	0.0049	VKORC1, DDHD1, NEDD4, CYP3A4
hsa-miR-183	0.0196	VKORC1, DDHD1, ASPH
hsa-miR-147b	0.0196	VKORC1
hsa-miR-1296	0.0196	VKORC1, CYP4F2
hsa-miR-1285	0.0049	VKORC1, DDHD1
hsa-miR-1207-5p	0.0196	CYP1A2, GGCX, DNMT3A, VKORC1, EPHX1
hsa-miR-1178	0.0196	VKORC1, NEDD4, CYP4F2, DDHD1, CYP1A1
hsa-miR-765	0.0001	HNF4A, DDHD1, THBD, GGCX, STX4, VKORC1, FPGS
hsa-miR-31	0.0196	NEDD4, VKORC1, FPGS, ASPH, CALU, DDHD1
hsa-miR-24-3p	0.0049	VKORC1, DDHD1, HNF4A, GGCX, ASPH
hsa-miR-1912	0.0196	NQO1, THBD, VKORC1, CALU
hsa-miR-1826	0.0196	HNF4A, ASPH, VKORC1
hsa-miR-1254	0.0196	VKORC1, DDHD1, NQO1, CYP1A1, EPHX1
hsa-miR-137	0.0049	ASPH, NR1I3, VKORC1
hsa-miR-133b	0.0049	VKORC1
hsa-miR-133a	0.0049	VKORC1
hsa-miR-1276	0.0196	CALU, VKORC1
hsa-miR-634	0.0137	FPGS, NQO1, HNF4A, VKORC1

### Validation study

We examined the three predicted miRNAs by hydrolysis probe-based RT-qPCR in the three dosage groups. Kruskal–Wallis test showed that hsa-miR-133b (*p* < 0.001) and hsa-miR-1276 (*p* = 0.024) were significantly different among the three groups, but hsa-miR-24-3p (*p* = 0.806) was not.

**Figure 3:**
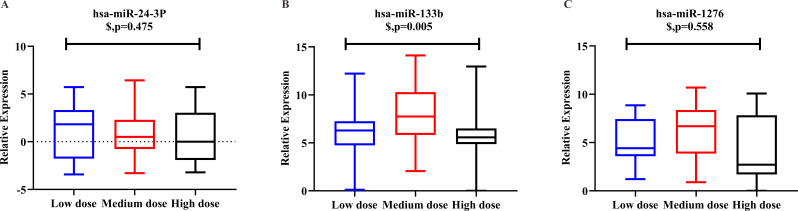
Relative expression level of hsa-miR-24-3p, hsa-miR-133b, hsa-miR-1276 among the three-dose groups. (A) hsa-miR-24-3p showed no significant differences in logistic regression results among the three-dose groups. (*p* = 0.475). (B) hsa-miR-133b showed significant differences in logistic regression results among the three-dose groups (*p* = 0.005). Furthermore, *t*-test among any two groups: there was a statistical difference between the “medium-dose” group and the “low-” and “high-dose” groups (*p* = 0.003, <0.001), but not between the “low-” and “high-dose” groups(*p* = 0.336). (C) hsa-miR-1276 showed no significant differences in logistic regression results among the three-dose groups (*p* = 0.558). $: *p*-value after correcting confounding factors by logistic regression (*p* < 0.05).

Logistic regression analysis was used to adjust for confounding factors. After adjusting for clinical covariates, including age, gender, height, weight, smoking habit, alcohol intake, and combination use of amiodarone, there were significant between-group differences among the three-dose groups for hsa-miR-133b expression (*p* = 0.005). However we observed an “n-shaped” dose-dependent curve rather than a linear relationship. There were no significant differences in hsa-miR-24-3p (*p* = 0.475) or hsa-miR-1276 (*p* = 0.558) among the three groups (Fig. 3).

## Discussion

Many factors can affect the warfarin effect, which brings significant challenges to determining precise individual dosages. An algorithm based on pharmacogenetic and clinical factors has been shown to be more helpful for determining stable warfarin dosages compared to the standard dosing setting approach (Hu & Yu, 2013).However, the existing warfarin dose prediction model still cannot meet clinical demands. Epigenetic pharmacologic markers could be used to guide individualized warfarin therapy; supplementing and improving the existing pharmacogenetic algorithms would like to improve drug cost-effectiveness (Yu, 2009). Roles of miRNAs in drug toxicity, safety assessment (Marrone, Beland & Pogribny, 2015), and regulation of drug metabolism and disposition (Yu, 2009) have been described. Previous studies have reported several miRNAs polymorphisms related to warfarin dose, and warfarin itself can also affect miRNAs expression (Ciccacci et al., 2015; Rad, Mazaheri & Firozabadi, 2018; Hosseindokht et al., 2020; Jia et al., 2015). These studies suggest that miRNAs may have great value as new biomarkers and therapeutic targets.

However, no investigation has been undertaken on specific miRNA profiles associated with stable warfarin dose. Chen and colleagues suggested that hsa-miR-24-3p could act on coagulation factor X and participate VKORC1 gene expression (Chen et al., 2017). We also found that hsa-miR-1276 and hsa-miR-133b could bind to the VKORC1 3′ UTR, which was consistent with previous results (Pérez-Andreu et al., 2013). Although VKORC1 mRNA levels are crucial to warfarin dosing, potential effects of miRNAs on VKORC1 gene expression during warfarin therapy should be further confirmed. Our results identified hsa-miR-133b as a possible reference factor for the warfarin dosage algorithm. However, hsa-miR-24-3p and hsa-miR-1276 showed no significant differences among the three groups on logistic regression analysis. There were significant between-group differences about hsa-miR-133b levels, but we observed an “n-shaped” dose-dependent curve rather than a linear relationship.

The drug dose–response relationship is often complicated. Jia et al. (2015) reported that relative hsa-miR-133b levels were significantly increased in patients taking oral warfarin who underwent cardiac valve replacement. This suggested that hsa-miR-133b could be a possible biomarker to guide personalized warfarin dosage. To better understand the “n-shaped” dose-dependent model, we searched PubMed using the terms “miR-133” AND “VKORC1” and “miR-133” AND “warfarin”. Hypothetically, hsa-miR-133b may participate in the normal physiologic response to warfarin. Besides, hsa-miR-133b also plays an essential role in heart injury repair (Liu et al., 2017) and drug toxicity (Marrone, Beland & Pogribny, 2015). One possible mechanism is that there is a negative feedback mechanism between hsa-miR-133b and the stable warfarin dose; that is, the intake of warfarin caused a first increase in the expression of hsa-miR-133b. Subsequently, hsa-miR-133b can downregulate VKORC1 expression by binding with VKORC1 3′ UTR, which indirectly reduces the body’s sensitivity to warfarin. In this scenario, a larger dose is required to achieve the desired anticoagulant effect, and a negative feedback mechanism is activated to restore responsiveness to warfarin, mainly through the negative regulation of hsa-miR-133b to weaken VKORC1 downregulation. This may be one possible mechanism for the “n-shaped” dose-dependent relationship in our findings.

The use of miRNAs as biomarkers to guide the drug dose adjustment must thoroughly investigated in epigenome-wide association studies. Specifically, the causal relationship between phenotype and miRNAs expression needs to be clarified. Before a single miRNA or a group of miRNAs can be used to guide dosages, researchers must mechanistically establish whether differential miRNAs lead to phenotypic alterations and vice versa. Furthermore, it must be determined if the decrease in relative hsa-miR-133b expression is a negative feedback regulation induced when the body recovers from warfarin tolerance to normal reactivity.

There were three main limitations of this study. (1) Although the dose groups were matched by baseline patient characteristics, the impacts of CYP2C9, VKORC1 and miR-133 genotypes were not considered and may be confounding factors. (2) The sample size and the number of target miRNAs were insufficient and may have decreased the statistical power. (3) Finally, the study population consists mainly of Han people in Fujian province, and extrapolation of study findings to other regions should be made with caution. We hope that future researches can further consider these factors based on our experience.

## Conclusion

In summary, miRNAs have received increasing attention as precision medicine biomarkers and therapeutic targets in various diseases. Our investigation revealed that hsa-miR-133b might be a new possible reference factor to improve the warfarin dosage algorithm. However, because of our small sample size and the complex relationship between miRNAs expression and warfarin dose, further prospective studies are needed. If the “n-shaped” dose-dependent curve is confirmed, miRNAs will be valuable biomarkers to guide warfarin precision medication. This study provides a new perspective, but no clear explanation and further work is needed to understand the importance of miRNAs in warfarin precision medicine. It emphasizes the importance of comprehensively evaluating complex relationships in warfarin dose prediction models and provides future pharmacogenetics research ideas.

##  Supplemental Information

10.7717/peerj.9995/supp-1 Supplemental Information 1 The relative expression of three target miRNAs Baseline characteristics were compared by the Kruskal–Wallis test (for continuous variables) or Fisher’s exact test (for categorical variables) to analyze differences among the three groups. The relative expression level of target miRNAs (denoted by Ct) was corrected by cel-miR-39(Ct_= Ct_-Ct_cel-miR-39) and the reference sample(Ct_= Ct_sample -Ct_ref. sample),and then used as the data for subsequent statistical analyses.

## Additional Information and Declarations

### Data Availability

The following information was supplied regarding data availability: The raw measurements are available as a Supplemental File.
